# Development of MEMS Process Compatible (Bi,Sb)_2_(Se,Te)_3_-Based Thin Films for Scalable Fabrication of Planar Micro-Thermoelectric Generators

**DOI:** 10.3390/mi13091459

**Published:** 2022-09-02

**Authors:** Prithu Bhatnagar, Daryoosh Vashaee

**Affiliations:** 1 Department of Electrical and Computer Engineering, NC State University, Raleigh, NC 27695, USA; 2 Department of Materials Science and Engineering, NC State University, Raleigh, NC 27695, USA

**Keywords:** thermoelectric generators, MEMS, thermoelectric thin films, thermal evaporation, bismuth telluride

## Abstract

Bismuth telluride-based thin films have been investigated as the active material in flexible and micro thermoelectric generators (TEGs) for near room-temperature energy harvesting applications. The latter is a class of compact printed circuit board compatible devices conceptualized for operation at low-temperature gradients to generate power for wireless sensor nodes (WSNs), the fundamental units of the Internet-of-Things (IoT). CMOS and MEMS compatible micro-TEGs require thin films that can be integrated into the fabrication flow without compromising their thermoelectric properties. We present results on the thermoelectric properties of (Bi,Sb)_2_(Se,Te)_3_ thin films deposited via thermal evaporation of ternary compound pellets on four-inch SiO_2_ substrates at room temperature. Thin-film compositions and post-deposition annealing parameters are optimized to achieve power factors of 2.75 mW m^−1^ K^−2^ and 0.59 mW m^−1^ K^−2^ for p-type and n-type thin films. The measurement setup is optimized to characterize the thin-film properties accurately. Thin-film adhesion is further tested and optimized on several substrates. Successful lift-off of p-type and n-type thin films is completed on the same wafer to create thermocouple patterns as per the target device design proving compatibility with the standard MEMS fabrication process.

## 1. Introduction

**Figure 1:**
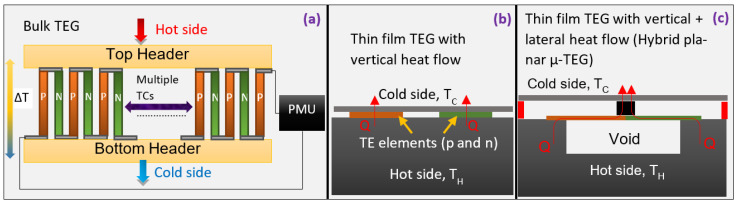
(**a**) Conventional bulk TEG design with p-type and n-type thermoelectric legs placed between two headers. (**b**) A thin film TEG with vertical heat flow. (**c**) A thin film TEG with vertical and lateral heat flow, classified as a hybrid planar TEG.

Driven by the demand for applications leveraging Internet-of-Things (IoT) networks [1] such as smart cities [2], industrial monitoring [3], smart homes [4], and health tracking wearables [5,6], the number of wireless sensor nodes (WSNs) was expected [1] to grow beyond 50 billion devices in 2020. However, the short lifespan of batteries and the several trillion devices envisaged has dampened expectations [7] such that the actual number of WSNs remained below 25 billion. Among efforts to decrease the power consumption of WSNs and increase the capacity and lifetime of batteries [8], research into energy harvesting systems [9] has been gaining importance over the last decade with a focus on four major technologies, including thermoelectric generators [10,11], piezoelectric generators [12], triboelectric generators [13], and compact solar cells [14]. Multi-modal solutions are likely to be the most optimum. Still, TEGs have their advantages of low maintenance due to the absence of moving parts, DC voltage output leading to simpler interfacing with power management units, reliable operation, and availability of temperature gradients in numerous application scenarios [10]. Bismuth telluride has been the material of choice in industry and research areas for harvesting heat around room temperature [11]. Commercial modules of TEGs utilize bismuth antimony telluride, Bi_2_(Se,Te)_3_, and bismuth selenium telluride, (Bi,Sb)_2_Te_3_, materials with zT of around 0.7 to 1 for n-type and p-type legs, respectively [15]. Here the thermoelectric figure-of-merit zT = S^2^σT/κ, where S is the Seebeck coefficient, σ is electrical conductivity, and κ is the thermal conductivity. Higher zT in the ranges of 1.2–1.4 has been achieved through the development of nanostructured (Bi,Sb)_2_Te_3_ [16] and Bi_2_(Se,Te)_3_ alloys [17]. When these nanocomposite materials have been integrated into small form factors for body heat harvesting [11], the output power is still insufficient to drive WSN platforms that consume power above 100 µW. The low-temperature gradients available for applications such as body heat harvesting and environmental monitoring increase the importance of managing the thermal resistances at the TEG-environment interface and motivate exploration of new device designs to maximize the thermal resistance (R_TH_) of the TEG itself. A device with a high R_TH_ boosts the temperature difference across the thermoelectric material, but there are limited avenues for increasing the R_TH_ of a conventional bulk TEG, as pictured in Figure 1a. By increasing the length of the legs, the R_TH_ of conventional TEGs can be raised but at the cost of device size and fragility from higher aspect ratios. Heat sinks have been effective in reducing the interface R_TH_ but can add bulk to the device, affecting user comfort in wearable applications. The need to overcome these limitations has fueled research [18] into hybrid heat flow micro-TEGs and flexible TEGs.

As demonstrated in Figure 1c and classified in [18], hybrid µ-TEGs are a device design that enables increasing the R_TH_ by forming membranes housing thermoelectric thin films. Since the heat flow across the thermoelectric is horizontal, the active elements can be elongated while maintaining a compact form factor. Unlike thin film TEGs with vertical heat flow (Figure 1b), expensive vacuum deposition of several 10′s of micron thick films is unnecessary. The fabrication of a hybrid µ-TEG is challenging due to the need to create vacuum cavities to eliminate parasitic thermal resistance. Such designs have been explored with CMOS [19] and MEMS-based [20] fabrication setups but with poly-Si and SiGe thin films. The MEMS-based approach relies on wafer bonding as a tool to create vacuum cavities on both sides of the thermoelectric thin films. Thus, demonstrating a hybrid planar TEG with bismuth telluride thin films requires developing a process where both horizontal (through the thin films) and vertical (through wafer bonding rings) heat flow paths can be created. This work reports results from the pre-packaging fabrication steps for a hybrid planar µ-TEG with bismuth telluride thin films. High-performing bismuth telluride-based thin films initially adopted for micro-cooling applications [21] are gaining relevance [22] for state-of-the-art generators operating at low-temperature gradients. Co-evaporation [23,24], co-sputtering [25,26], molecular beam epitaxy [27,28], pulsed laser deposition [29,30], and flash evaporation [31,32] are some techniques that have been explored widely. Among them, co-evaporation has been part of attempts at making devices [21,33] with micro-patterned features. However, the best quality films via co-evaporation are achieved at elevated substrate temperatures [22] above 250 °C. This causes complications while using photoresists to pattern features or deposits on common flexible substrates like PET as they can get burnt and stick to the wafer. Researchers have explored methods such as co-sputtering followed by annealing [34], thermal diffusion [35], and working with lift-off resists that have a high-temperature reflow point [36]. In this work, we explore room temperature evaporation from ternary compound pellets of Bi_2-x_Sb_x_Te_3_ and Bi_2_Se_x_Te_3-x_ and pattern the films as per the design of a hybrid planar µ-TEG. This method alleviates the challenges of co-evaporation and MEMS process incompatibilities due to high substrate temperatures.

## 2. Experimental

Thermoelectric thin films are deposited on different substrates by evaporating ternary compound pellets at room temperature. After initial optimization of the film compositions, we demonstrate lift-off and subsequently integrate the films into the first three stages of fabricating a planar hybrid µ-TEG.

**Figure 2:**
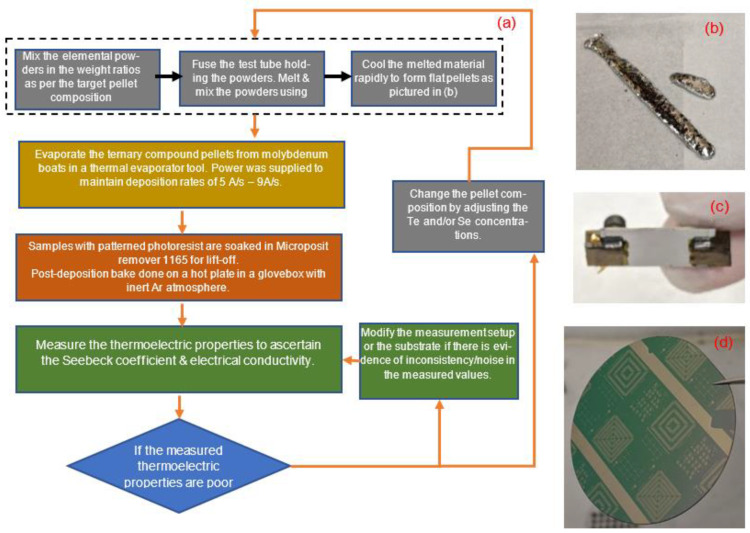
(**a**) The flowchart details the steps to achieve device-quality thin films. The composition of the ternary compound pellets and the post-deposition anneal were the key parameters explored for optimization. (**b**) Pellets evaporated to form thermoelectric thin films. (**c**) A 17 mm × 8 mm thin film sample mounted for measurement on the LSR tool. (**d**) Thermoelectric thin films patterned via lift-off process.

Ingot synthesis follows the previously reported process [37]. In brief, the process (Figure 2a) started with the synthesis of the (Bi,Sb)_2_(Se,Te)_3_ pellets. In a glovebox with an inert argon environment, we mixed elemental powders (99.99% purity) of Bi, Sb, and Te for p-type pellets, as per the target stoichiometric ratios, transferred them into a quartz tube, and sealed the tube. The tube was then transferred out of the glovebox and heated over an acetylene torch to thoroughly melt and mix the elements. This melted mixture was then quenched and solidified into pellets. A similar procedure was followed to synthesize the n-type pellets with Bi, Te, and Se powders. Before deposition in the thermal evaporator tool, the substrates were diced or cut with a wire saw into 17 mm × 8 mm pieces to ensure compatibility with the thermoelectric measurement setup and then cleaned with acetone and isopropyl alcohol. The pellets were loaded into molybdenum boats and evaporated onto the substrate, with the deposition rate varying from 5 Å/s to 9 Å/s. Chamber pressure was maintained at 7.5 × 10^−6^ Torr. The substrate was rotated but not heated. After deposition, the samples were transferred to a glovebox and heated over a hotplate at temperatures varying from 260 °C to 360 °C. The thermoelectric properties of the samples were measured in a Linseis LSR tool. The film thicknesses were measured with a Dektak Stylus profilometer, and the samples in this study varied in thickness from 1 µm to 2 µm. X-ray diffraction data from the thin films were obtained in a Rigaku Miniflex600 tool equipped with Cu-Kα radiation source. Lift-off of thermoelectric thin films was tuned on 2” Si/SiO_2_ wafers. A separate batch of samples consisting of 4” Si/SiO_2_ substrates was optimized for the microfabrication process consisting of metal deposition, electroplating, and thin film deposition. Those details are presented in Section 3.4.

## 3. Results and Discussion

### 3.1. Optimization of Thin Film Thermoelectric Properties

The targeted film compositions were Bi_0.5_Sb_1.5_Te_3_ for p-type thin films and Bi_2_Se_0.3_Te_2.7_ for n-type thin films, as some of the best room temperature zT data have been measured on similar bulk nanocomposite materials [16,17,38,39,40]. Pellet compositions per these target thin film stoichiometries were our initial starting points but were modified to increase the power factor, as discussed below. A difference in the stoichiometry of the deposited thin films and the pellets is expected as the vapor pressures of the constituent elements are very different (Table 1). Substrates are typically heated to around 250 °C during deposition, which can cause re-sublimation of tellurium [33] (200 °C at 1.0 × 10^−5^ torr). Hence, co-evaporation works [23,24,41] have explored different flux ratios, and it was found that a Te:Bi ratio above the target stoichiometry is required for significant power factors. Since we evaporated from a single source, the pellet composition was tuned rather than the flux ratios as reported in works on co-evaporation. Disproportionate loss of materials during the post-deposition bake that promotes grain growth was countered by compensating for the loss at the pellet synthesis stage. The temperatures corresponding to the vapor pressure of 0.76 Torr as in the case of Te (505 °C) and Se (344 °C) compared with Bi (893 °C) and Sb (886 °C at 1 Torr), as listed in Table 1, underline the need for increasing the concentration of Se and Te in the pellets while mixing the materials. The volatile nature of Se was found to be a significant factor driving the tuning of pellet compositions. Using three examples, Table 2 shows the weight of materials used to prepare Bi_2_Se_0.3_Te_2.7_ pellets with different compositions for the optimization experiments.

**Figure 3:**
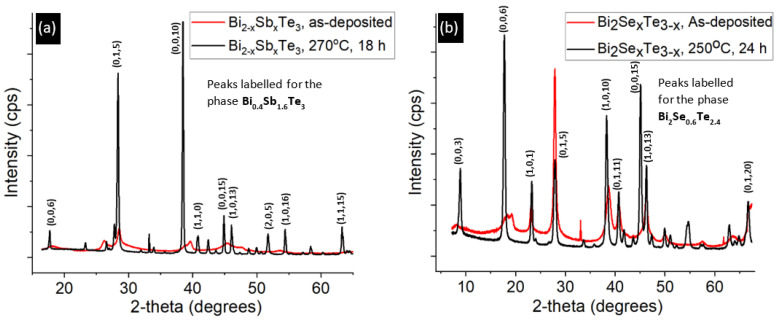
XRD data before and after post-deposition annealing. (**a**) P−type thin film sample demonstrates that baking at 270 °C for 18 h is essential for improving the crystallinity of the as−deposited film. (**b**) A similar change in crystallinity was observed on an n-type sample baked at 250 °C for 24 h.

Our nomenclature for referring to the samples describes the factor by which each element’s weight is more than the weight that would be assigned for the target composition. For example, Bi_2_Se_0.3_Te_2.7_ (Se6x, Te2x) implies that the weight of Se and Te are six times and two times the value that would be mixed to achieve the proportions as implied by the chemical formula Bi_2_Se_0.3_Te_2.7_. The second important parameter requiring optimization was the post-deposition anneal temperature and duration since, without substrate heating, the as-deposited films are partially amorphous, and annealing helps increase the crystallinity as well as improve the thermoelectric properties, as illustrated by the data in Figure 3 which correspond to a p-type sample with pellet composition Bi_0.5_Sb_1.5_Te_3_ (Te4x). The (0,1,5) plane is the second most dominant peak, concurring with reports of being dominant in thin films grown at low substrate temperatures and high growth rates [22], as in this study. The n-type thin film data is displayed in Figure 3b with the peaks marked for the phase Bi_2_Se_0.6_Te_2.4_. The phases are identified using the PDXL software by Rigaku (Tokyo, Japan) that refers to the Crystallography Open database.

**Figure 4:**
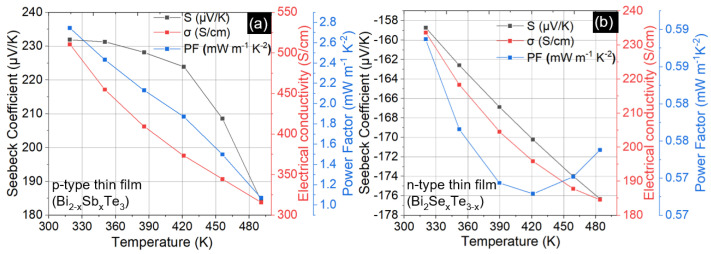
Thermoelectric properties versus temperatures for p-type (**a**) and n-type (**b**) films deposited on glass substrates. Amongst the samples we synthesized, these films had the highest power factor near room temperature.

**Figure 5:**
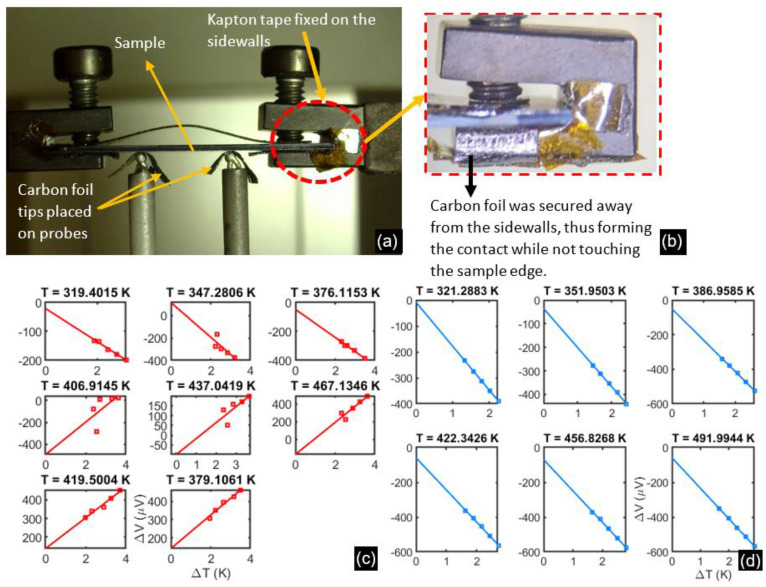
(**a**) Thin film samples are placed upside down and secured by screws on the end supports. The left support heats up, creating a temperature gradient measured by the thermocouple probes shown. These probes also document the Seebeck voltage across the probe distance, which was ~3.5 mm. (**b**) The inset shows that the carbon foil and Kaptan tape were added to prevent substrate conduction. (**c**) Noisy and inconclusive voltage-temperature gradients were measured before modification of the setup. (**d**) Repeatable and good quality data with the upgraded setup. Both (**c**,**d**) were measured on thin films deposited on Si/SiO_2_ substrates.

Figure 4 presents data from a p-type and an n-type thin film deposited on glass substrate to outline the scope of our thermoelectric measurements on the LSR tool. At each temperature, the Seebeck coefficient and electrical conductivity were calculated over four separate voltage-temperature data sets consisting of five data points, i.e., a total of twenty measurements for each temperature point, with the average taken as the value for that temperature. Figure 5d shows the averaged data and the line fit to find the Seebeck coefficient, i.e., the slope of the line. The power factor (PF) was then calculated as per the definition PF = S^2^σ where S is the Seebeck coefficient and σ is the electrical conductivity. The data in Figure 4 represents some of our best samples attained after the optimization process discussed in the remainder of this section. Power factors of 2.75 mW m^−1^ K^−2^ and 0.59 mW m^−1^ K^−2^ are observed near room temperature and the power factor is seen to decrease with increasing temperature, largely because of the reduction in electrical conductivity. Data from other optimized samples on different substrates are provided in Table 3 in Section 3.3.

The first batch of samples was deposited on Si/SiO_2_ substrates to replicate the final device requirements. Still, erroneous voltage-temperature data were observed, likely due to inadvertent substrate conduction, as shown in Figure 5c. To circumvent this, we used glass substrates, and the measurement setup was modified in Figure 5b so that the sidewalls of the sample holders were never in contact with the sample substrate. Adopting these changes improves the repeatability of the voltage-temperature gradients on subsequent Si/SiO_2_ substrates, as seen in Figure 5d. Additionally, non-conductive glass substrates serve as essential control samples to guide the optimization of the thermoelectric thin films.

**Figure 6:**
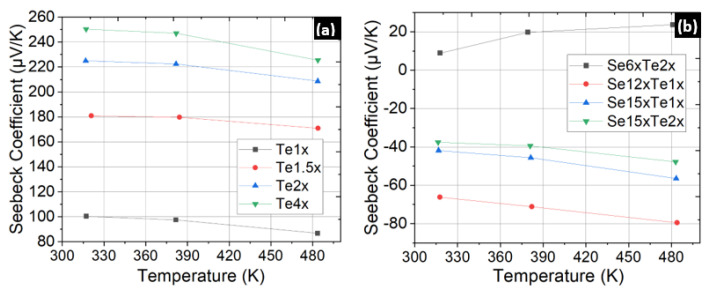
(**a**) Power factor of p-type thin films showing the correlation between the Te composition in the pellets and the thermoelectric performance. All samples were baked for 24 h at 250 °C before measurement. (**b**) Seebeck coefficient measured for n-type thin films prepared with the pellet compositions stated in the legend. Poor quality n-type thin films with Seebeck coefficients below −80 µV/K were observed, and a mildly positive S was seen for the Se6xTe2x case.

Pellet compositions and annealing parameters were optimized iteratively until a power factor that can enable acceptable device performance was measured. It was found that for p-type thin films, a composition with Te being 2x to 4x times the weight according to the stoichiometric ratio yielded the highest power factor. Figure 6a shows the power factor enhancement when the pellet composition is changed from Te1x to Te4x. In the case of n-type thin films, initial samples with Se and Te set to 2x to 6x times the corresponding stoichiometric weights failed even to register a negative Seebeck coefficient. A mildly negative Seebeck was finally measured upon increasing the Se concentration to form thin films by evaporating Se12xTe2x pellets. The bake parameters were kept the same as those for the p-type thin films, i.e., 250 °C for 24 h. Since the power factor was less than 0.3 mW m^−1^ K^−2^, we evaporated thin films from Se15xTe (1x-2x) pellets, but the Seebeck coefficient was seen to decrease by 30% (Figure 6b).

**Figure 7:**
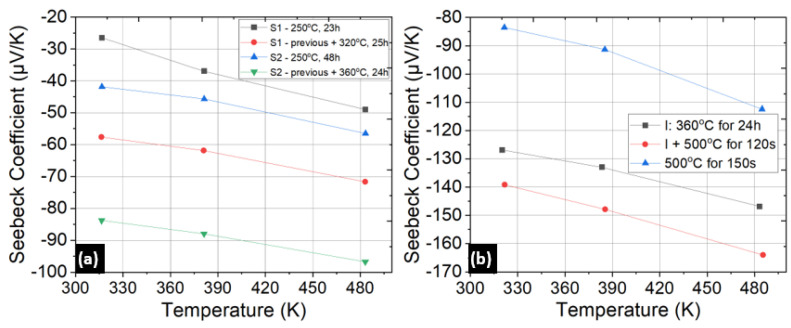
(**a**) Increase in the Seebeck coefficient of n-type Se12xTe1x thin films seen upon long bakes at higher temperatures of 320 °C (Sample 1 –> S1) and 360 °C (Sample 2 –> S2). (**b**) Seebeck coefficients of the optimum thin films obtained from pellet composition of Se9xTe2x, and a high-temperature bake at 360 °C. Short-term high heat treatment at 500 °C showed improvement but was also seen to damage the surface of one of the samples; hence, it was excluded from the subsequent batches.

A 100% improvement in the Seebeck coefficient and power factor of n-type thin films was attained upon increasing the post-deposition bake temperatures to 320 °C and 360 °C, as depicted in Figure 7a. The optimum recipe for n-type thin films was finalized after measurements on thin films deposited from Se9xTe2x pellet composition and baked at 360 °C for 24 h. Samples from the Se9xTe2x composition showed a Seebeck coefficient of −130 µV/K at room temperature (Figure 7b). An increase of the Seebeck to −140 µV/K was observed after performing a rapid thermal treatment by leaving the sample on a hotplate that had been heated to 500 °C for 2–3 min. However, this treatment was not adopted for the final device films as the improvements were erratic (Sample 2 in Figure 7b) and could lead to surface damage. After determining the optimum pellet compositions, a final batch of samples was prepared for the lift-off and adhesion layer tests.

### 3.2. Lift-Off of Thermoelectric Thin Films

**Figure 8:**
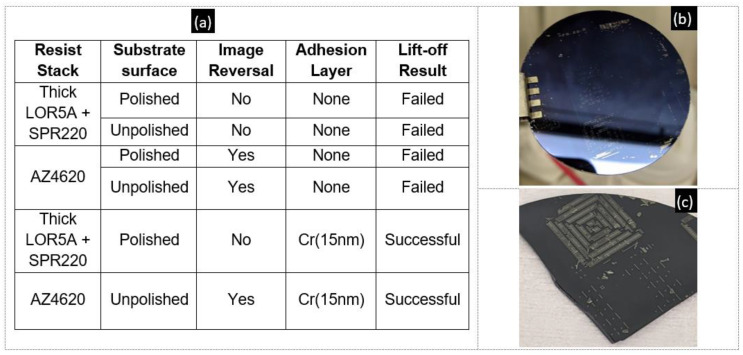
(**a**) Table tracking the combinations of adhesion layers, resists, and substrate surfaces tested for lift-off. (**b**) Failed lift-off on a polished surface of a 2” Si/SiO_2_ wafer. (**c**) Failed lift-off on an unpolished surface.

The lack of anisotropic wet etching methods and incompatibility of bismuth telluride compounds in dry etching tools at shared facilities strengthens the case for investigating lift-off. Shadow masks have been used [33] to pattern feature sizes down to 23 µm, and they also allow high-temperature deposition. However, the lift-off technique is more accessible given the standard lithography tools available in most research and industrial facilities and does not require custom equipment or alignment techniques. In parallel with tuning the thermoelectric properties of the thin films, we examined several approaches for lift-off. Image reversal with AZ4620 (Microchemicals, Baden-Wuerttemberg, Germany) resist and using the thick LOR5A/SPR220 (Kayaku advanced materials, Westborough, MA, USA) resist stack were the two tested lithography methods. Considering the requirement to deposit up to 2 µm thick thermoelectric layers, the latter involved four dispenses of LOR5A to create a 2 µm thick undercut layer. The AZ4620 resist was spun to create a 9 µm thick layer. Upon image reversal, an undercut exceeding 1 µm was observed. Patterns consisting of thermocouple dimensions of 400 µm × 100 µm were fabricated using the above methods on both polished and unpolished Si/SiO_2_ substrates where the SiO_2_ was a thermally grown 300 nm oxide layer. Figure 8a summarizes the tests.

**Figure 9:**
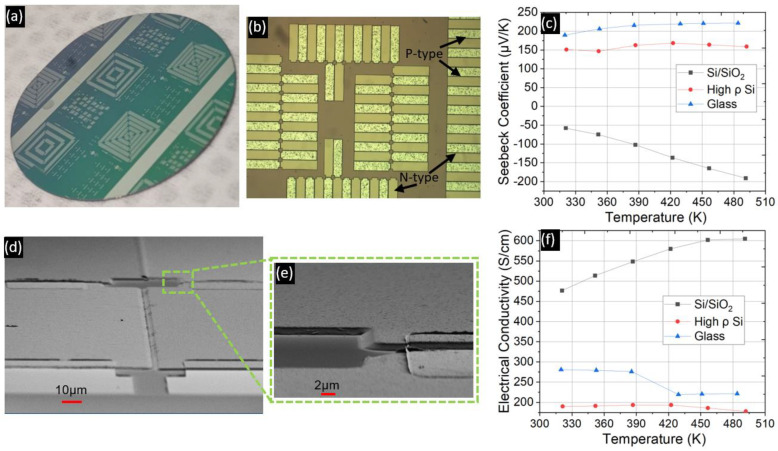
(**a**) Successful lift-off of p-type and n-type thin films on a 2” Si/SiO_2_ wafer. N-type thin films were deposited before p-type, requiring a higher post-deposition bake temperature. A 15 nm Cr adhesion layer was deposited in situ before the thermoelectric thin film evaporation. (**b**) Zoomed-in view of the wafer from (**a**). (**c**) Negative Seebeck coefficients were measured on a Si/SiO_2_ substrate where a Cr adhesion layer was deposited before evaporation of the pellets. The data with a positive Seebeck coefficient corresponds to samples loaded during the same run but on glass and high resistivity (ρ) Si substrates. (**f**) Corresponding electrical conductivity data. (**d**) SEM images showing the thermocouples displayed in (**a**,**b**). Ni contacts deposited over the thermoelectric thin films to form test structures are also visible (**e**) The zoomed-in view shows the slope of the thermoelectric thin films indicating that the rotation of the wafer can distort the boundary of the features and highlighting the importance of a large undercut during the lithography.

Test runs without depositing any adhesion layer largely failed irrespective of the chosen lithography technique or substrate, as summarized in Figure 8a. Cr has been reported [43] to improve the adhesion of Sb_2_Te_3_ thin films, which worked during the preliminary lift-off tests. It was found that a 15 nm Cr adhesion layer was necessary for good adhesion of the films during lift-off for both p-type and n-type thin films. Both the image reversal and the LOR-based lithography methods worked on patterning up to 2 µm thick films on the polished side of the Si/SiO_2_ substrates with a thin Cr adhesion layer. Adhesion of the films was not affected by the post-deposition anneal. Figure 9a,b,d, and e display lift-off results on a 2” Si/SiO_2_ substrate.

Although a thin 15 nm Cr adhesion layer facilitated lift-off of the thermoelectric thin films, measurement of samples on the LSR tool revealed peculiar behavior on p-type thin films deposited on Si/SiO_2_ substrates, where a Si/SiO_2_/Cr/Bi_0.5_Sb_1.5_Te_3_ stack of layers was formed. Such samples exhibited a negative Seebeck coefficient despite the measurement of a positive Seebeck coefficient on accompanying samples from the same run but with glass and high resistivity (ρ) Si pieces as substrates (Figure 9c).

The previous works [44,45] on using a Cr interlayer to promote adhesion of (Bi,Sb)_2_Te_3_ thin films to the substrate have reported an increase of 4x and 7x in the Seebeck coefficient compared to films without a Cr interlayer. Hence, our results were unexpected, although we note that the improvement in the Seebeck coefficient was reported for Cr interlayers that were 50 nm and 46 nm, respectively, whereas we deposited 15 nm Cr adhesion layers. Also, only [45] deposited thin films on Si/SiO_2,_ whereas in [44] the deposition was done on glass substrates. A study [46] comparing the effects of 1 nm and 1 µm of Cr layer on power generation of (Bi,Sb)_2_(Te,Se)_3_ thin films in a lateral heat flow thermoelectric module found that the thin films with a 1 µm Cr interlayer had Seebeck coefficients between 10 µV/K and −5 µV/K. The authors reported the formation of the Cr_2_Te_3_ phase from the XRD data. This study was done on SiO_2_ glass substrates rather than Si/SiO_2_.

Furthermore, as seen in Figure 9f, the electrical conductivity of the Si/SiO_2_/Cr/Bi_0.5_Sb_1.5_Te_3_ sample increases with temperature in contrast to the decreasing trends measured for the other substrates. Given that the thermal energy (k_B_T) available at 300 K is 0.026 eV and thus insufficient to excite carriers across the small bandgap of BiSbTe, we expect to see a decreasing trend, as in the case of high resistivity and glass substrates, assuming that phonon-induced carrier scattering is dominant.

Therefore, the ambiguity about the performance of the p-type thin films on Si/SiO_2_ substrates with a Cr adhesion layer, which is beyond the scope of this work, led us to explore another adhesion layer, as the following section elaborates.

### 3.3. Alternative Adhesion Layer

Since the planar TEG device was to be fabricated on Si/SiO_2_ substrates, and the Cr layer affected the properties of the p-type film, 3-mercaptopropyl trimethoxysilane (MPTMS) monolayer was investigated as an alternate adhesion layer. Poor adhesion of Au to glass drove efforts to investigate monolayers such as MPTMS, which have eliminated some disrupting effects of metallic adhesion layers for plasmonic applications [47]. The effect of MPTMS on the adhesion of Au to SiO_2_ substrates has also been explored [48,49]. It was found that the silane (Si-OCH_3_) group in MPTMS forms stable Si-O-Si covalent bonds on hydroxylated SiO_2_ thin films, leaving the thiol (SH) group on the opposite end [48,49]. MPTMS has also been employed as an adhesion layer for self-assembled Bi_2_S_3_ nanorods [50] and had been part of a study that showed enhanced electrical conductance at Cu-Bi_2_Te_3_ and Ni-Bi_2_Te_3_ interfaces. One study [51] postulated that MPTMS is chemisorbed on the Bi_2_Te_3_ surface with the thiol group anchoring the Bi_2_Te_3_ thin film. Hence, in the present work, the aim was to form a thiol-terminated MPTMS monolayer on SiO_2_ to promote adhesion to Bi_x_Sb_2-x_Te_3_ evaporated thin films. Following the procedure outlined in [51], MPTMS was applied to the Si/SiO_2_ substrate by soaking the substrate in a 10 mM solution of MPTMS in toluene, followed by a rinse in toluene and drying by a nitrogen gun to remove the excess MPTMS molecules.

Table 3 displays thermoelectric measurement data from several p-type and n-type samples at room temperature and 150 °C on different substrates with different adhesion layers. Pellet composition and post-deposition bake parameters were fixed based on previous tests. Along with glass and Si/SiO_2_ substrates, we loaded high resistivity (ρ) Si pieces which were diced from an undoped <100> 4” Si wafer having a resistivity greater than Ohm cm. For each of the runs in Table 3, full or half 2” wafers patterned with the image reversed AZ4620 resist were also loaded to test lift-off.

**Figure 10:**
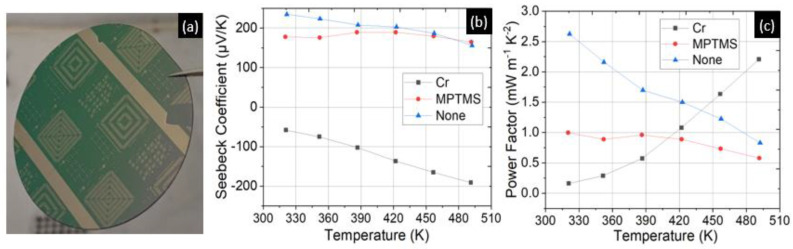
(**a**) Successful lift-off on a Si/SiO_2_ substrate after deposition of p−type thin films. (**b**,**c**) Comparison of thermoelectric data measured on Si/SiO_2_ substrates from runs 1r, 1s, and 1t from Table 3 with the adhesion layers as stated in the legend. Application of MPTMS resolved the negative Seebeck data issue while facilitating lift-off of p-type thin films.

The results from Table 3 were vital in designing the device fabrication process. Firstly, successful lift-off of p-type thin films was observed on Si/SiO_2_ substrates treated with MPTMS (Figure 10a). Secondly, the application of MPTMS did not lead to negative Seebeck data as was seen with a Cr adhesion layer (Figure 10b,c). The data reported in Table 3 were measured on 17 mm × 8 mm substrates loaded together with the 2” wafers set aside for lift-off tests. Although lift-off was achieved, the thermoelectric performance of p-type thin films (Runs 1r and 1s) deteriorated compared with results from the run without any adhesion layer (Run 1t from Table 3). Furthermore, the data shows that the best power factors from each run were measured on the glass substrates, irrespective of the adhesion layer.

Based on the data from glass and high resistivity Si samples, we did not see an increase in the Seebeck coefficient, whereas on the SiO_2_ substrate a negative Seebeck was measured. An error in the measurement setup is unlikely because, when comparing samples from the same run but on different substrates, we measured a positive Seebeck as expected, and the data from Run 1s with MPTMS on SiO_2_ thin film indicated a p-type thermoelectric behavior. Results from several works on room temperature deposition of (Bi,Sb)_2_(Se,Te)_3_ thin films are presented in Table 4. 

### 3.4. Results from Pre-Packaging Fabrication Steps

**Figure 11:**
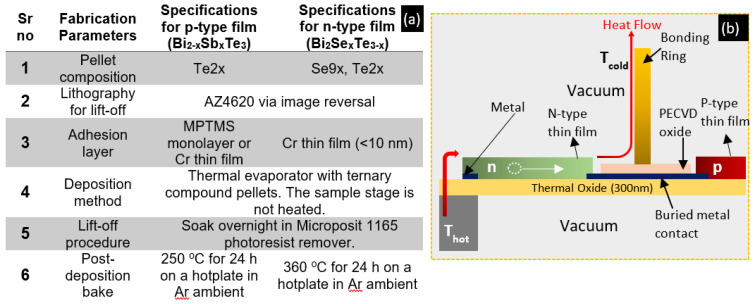
(**a**) Table showing the parameters used for the thermoelectric thin film deposition after optimization. (**b**) Cross-sectional schematic view of a hybrid heat flow planar TEG excluding the top and bottom headers.

Following the optimization of the thin films and their lift-off, they were integrated into the pre-packaging fabrication process for a planar TEG with a hybrid heat flow. Figure 11b shows a single thermocouple representing the heat flow in horizontal and vertical directions. The device we are attempting to fabricate consists of several hundred thermocouples arranged in rings so that their voltages add up. Since our focus is only on process compatibility and this device fabrication is in progress, we have not discussed the current paths. The pre-packaging process can be divided into three stages—deposition of interconnects, formation of bonding rings, and evaporation of thermoelectric thin films via lift-off. Deposition and bake parameters for optimized thin films (data reported in Figure 4) are summarized in Figure 11a.

The three stages of fabrication required six rounds of lithography. The exposure and alignment were completed in a Suss MicroTec (Garching, Germany) MA6 contact aligner. The parameters presented in Figure 11a were deployed during the thin film deposition stage. To minimize the exposure of the thermoelectric thin films to high temperatures, they were deposited towards the latter stages of the pre-packaging device process.

**Figure 12:**
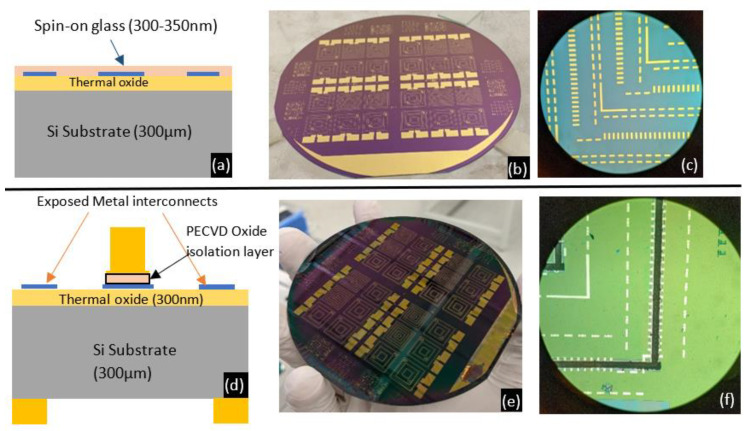
(**a**) Schematic view of the wafer at the end of Stage 1. (**b**) 4” wafer after Stage 1 with Ti/Ni/Au interconnects. (**c**) Zoomed-in view of the interconnects. (**d**) Schematic view after Stage 2 of fabrication. (**e**) Wafer-level view post Stage 2 after completing bonding ring formation on both sides of the wafer followed by etching of the PECVD oxide to reveal the metal interconnects. (**f**) Zoomed-in top view of the wafer at the end of Stage 2.

Stage 1 involved the formation of Ti (15 nm)/Ni (150 nm)/Au (100 nm) interconnects on 4”, 300 µm thick Si/SiO_2_ wafers (Figure 12a–c) where the SiO_2_ layer is a thermally grown oxide that is 300 nm thick. The interconnects were deposited by e-beam evaporation and were coated with a plasma-enhanced chemical vapor deposition (PECVD) oxide layer deposited at 200 °C to form a protective platform over the interconnects during Stage 2 of fabrication. Piranha cleaning (3:1 ratio of 96% sulphuric acid to hydrogen peroxide) of the substrates prior to interconnect deposition was found to be integral in protecting the interconnects against damage from thermal shock during the PECVD oxide deposition. This thermal shock was seen to cause the contacts to peel off when piranha cleaning was not used.

The objective of Stage 2 was to form Au bonding rings over selected areas. These bonding rings would later be capped with Sn to enable the formation of eutectic Au-Sn bonds and thus seal the cavity. Approximately 5 µm high bonding rings were deposited by electroplating on both sides of the wafer (Figure 12d–f). Image reversed AZ4620 resist was used as a mold during electroplating. A cyanide-free Au electroplating solution (24k Pure Eco by Gold Plating Services) worked well to form the bonding rings. However, the high pH of the solution could lead to delamination of the photoresist. Therefore, a Cr adhesion layer was deposited over the Au seed layer, an image reversal step was included, and the resist was hard baked before electroplating. Figure 12 shows the progress over these two stages. At the end of Stage 2, the protective PECVD oxide was etched away in a reactive ion etch system with a CHF_3_-based plasma to reveal the metal interconnects. AZ4620 resist was patterned to act as the etch mask over the bonding rings on the top side of the wafer.

**Figure 13:**
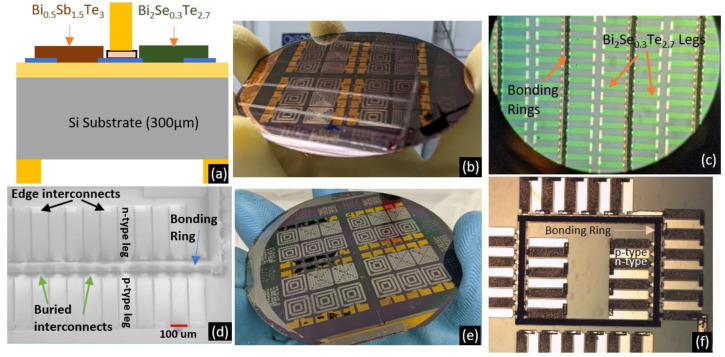
(**a**) Schematic view of the cross-section of one thermocouple after Stage 3 of fabrication. (**b**) Wafer view after evaporation and lift-off of n-type thin films. (**c**) Zoomed-in view of n-type thin films. (**d**) SEM image of a section of a single device after deposition of both p-type and n-type thermoelectric thin films. (**e**) Wafer view after both rounds of deposition. (**f**) Zoomed-in picture of a section of the device.

The wafer was now ready for deposition of thermoelectric thin films by thermal evaporation from ternary compound pellets, i.e., Stage 3. After lithography to define the thermocouple patterns, n-type thin films using Se9xTe2x pellets were evaporated, the wafer was left for lift-off in Microposit 1165 (Dow electronics, Tampa, FL, USA) remover, and then the post-deposition bake was done as per the parameters in Figure 11a. N-type deposition was completed first as the post-deposition bake temperature required 360 °C, substantially higher than the 270 °C required for the p-type thin films. The p-type thin films had shown surface damage when baked at 360 °C whereas the n-type thin films remained unaffected during a bake at 270 °C following their initial bake at 360 °C. The aim was to create a cross-section as displayed in Figure 13a. Following n-type thin film deposition and lift-off, similar steps were followed for p-type thin films with details as presented in Figure 11a. After Stage 2, the topology of the wafer had significant height differences due to the 5 µm bonding rings, so the lithography for the thin films’ lift-off was done using image reversed AZ4620 resist that was 9 µm thick and hence could effectively coat the 5 µm bonding rings. MPTMS and Cr thin films acted as the adhesion layers during processing p-type and n-type thin films, respectively.

Delaying the deposition of the thermoelectric thin films until the point after the bonding rings formation has multiple benefits. It avoids accidental exposure of the films to electroplating solution when forming the backside bonding rings. Since the formation of bonding rings involves exposure of the wafer to both the Cr etch and Au etch solutions, any thermoelectric thin films present without requisite shielding might sustain damage. Metal interconnects deposited in Stage 1 were shielded by the PECVD oxide but we did notice damage to these interconnects if there were pinholes due to a poor quality oxide. This was one of the reasons behind not attempting deposition of oxide at lower temperatures or using less costly alternatives such as spin-on glass, as the oxide would have been less dense. Successful coating of oxide or alternative protective layers on thermoelectric thin films would still create the complication of dry etching the protective layer without damaging or exposing the thermoelectric thin films to the plasma as that can cause cross-contamination in shared facilities. The uneven topology of the wafer with BiTe thin films would have made it very difficult to deposit a continuous seed layer for electroplating. Finally, by preponing the interconnects and bonding rings formation, all metal interconnects could be deposited in a single run rather than the edge contacts being deposited after the thin film evaporation. This reduced the number of lithography masks to be fabricated.

We now compare results after Stage 3 from two wafers that revealed intriguing trade-offs between utilizing MPTMS and Cr as adhesion layers for the p-type thin films. The first wafer (W1) was prepared with Cr (10 nm) as the adhesion layer for n-type thin films and MPTMS for anchoring the p-type thin film to the substrate. The adhesion of n-type thin films to the Cr interlayer failed in several areas on the wafer. The p-type thin films with an MPTMS adhesion layer showed improved adhesion, but around 30% of the thermocouples had signs of partial delamination. On the other hand, wafer 2 (W2) utilized Cr (10 nm) as the adhesion layer for both p-type and n-type thin films. Lift-off was successful for both rounds of thermoelectric thin film evaporation in W2. Results from W2 after Stage 3 of fabrication are depicted in Figure 13.

**Figure 14:**
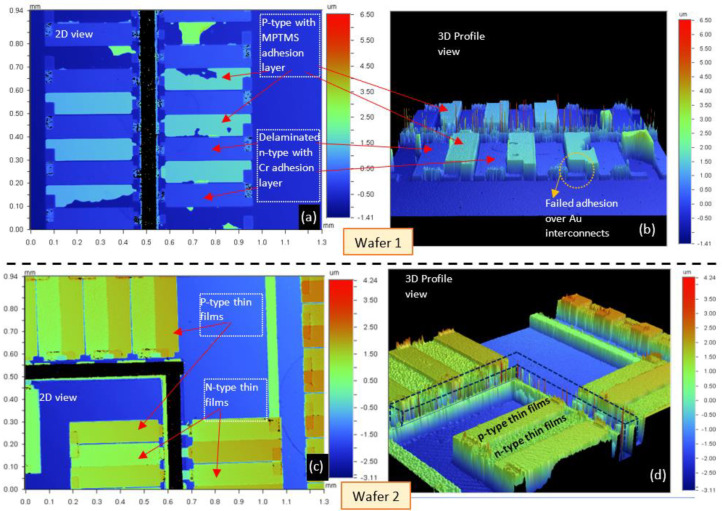
(**a**) 2D view of a section on W1 depicting the failed lift−off of n−type thin films having a Cr adhesion layer and the partially failed lift−off of p−type thin films with MPTMS adhesion layer (**b**) 3D profiles created from (**a**) that highlight the lack of adhesion between p−type thin films and the interconnects. In the n−type areas, only the Cr adhesion layer is left. (**c**) 2D view of a section on W2 displaying the thermoelectric thin films showing the p−type and n−type thin films, metal contacts, and the bonding ring. (**d**) 3D view created from the optical profilometry data measured in (**a**). The dashed lines are drawn to indicate the bonding ring as the profilometry tool could not focus on the top of the Au bonding ring surface.

Unlike W1, we saw approximately less than 5% of thermocouples delaminate in W2, the majority of them being n-type thin films. Although on W2 we achieved complete thermocouple patterns for 50% of the 24 devices visible in Figure 13e, it required us to use Cr as the adhesion layer for both p-type and n-type thin films. Despite the Cr adhesion layer being reduced from 15 nm during test runs to 10 nm for W1 and W2, the lift-off worked for W2, so the thickness reduction in Cr layer is probably not the reason for failure in W1. Considering the increase in the size of the samples compared to test runs, the post-deposition bake for W1 after n-type deposition had been carried out at 400 °C instead of 360 °C. For W2, the temperature was set back to 360 °C. Hence, the higher temperatures during the bake for W1 may have led to the deterioration of the adhesion between the Cr interlayer and the n-type Bi_2_Se_x_Te_3-x_ thin films. Figure 14 uses optical profilometry data measured on a Wyko NT9100 Optical Profilometer to create 3D profiles of areas on W1 and W2.

Figure 14a,b illustrate that on W2, complete delamination of the n-type thin films was observed, leaving behind only the Cr adhesion layer. The MPTMS also did not promote adhesion between Au and p-type thin films, as marked on Figure 14b. A possible explanation is that since both Au and Bi_x_Sb_2-x_Te_3_ have been shown to be anchored via the thiol group on MPTMS, creating an Au/MPTMS/ Bi_x_Sb_2-x_Te_3_ stack presents a conflict. Replacing Au with another metal or depositing edge contacts on top of the thin films rather than below them are two measures that could circumvent this issue.

The optical profilometry images from Figure 14c,d provide a 3D view of the wafer (W2) shown in Figure 13. The dark region in Figure 14c represents the Au bonding ring. In Figure 14d, we draw dashed lines to show the bonding ring topology. The ability to reproduce such structures on a wafer scale is an essential milestone in our objective of fabricating a hybrid planar µ-TEG with (Bi,Sb)_2_(Te,Se)_3_ thin films. As fabrication progresses to subsequent stages, we anticipate further changes depending on the device characterization data. It is also observed that p-type thin films have a greater surface roughness than n-type thin films. Thus, comparing results from W1 and W2 reveal some interesting trade-offs. Using a Cr adhesion layer for p-type and n-type thin films enables the formation of the desired topology but can present a performance trade-odd since we have measured negative Seebeck coefficients on Si/SiO_2_ samples as discussed in Section 3.2. On the other hand, MPTMS-anchored Bi_x_Sb_2-x_Te_3_ thin films show a positive Seebeck coefficient on Si/SiO_2_ substrates, but on a wafer level (W1) the adhesion is not as consistent as that for W2. The lack of adhesion to edge interconnects that have Au as the top layer is also a concern. There are two sets of changes that may help. Firstly, we can repeat W1′s processing but with a reduced post-bake temperature for n-type thin films to enable lift-off and shift to the edge metal interconnects on top of the thin films to avoid complications with Au contacts. Secondly, the Si/SiO_2_ substrates could be replaced with high resistivity Si substrates as that would allow us to utilize a Cr adhesion layer for both the rounds of evaporation while also avoiding the counter-intuitive thermoelectric performance of the Si/SiO_2_/Cr/Bi_x_Sb_2-x_Te_3_ stack. The creation of vacuum cavities below the thin films will require an etch stop layer with high selectivity with Si during deep reactive ion etching (DRIE). Hence, we intend to look at silicon-on-insulator (SOI) wafers with highly insulating Si as the top layer for device fabrication.

**Figure 15:**
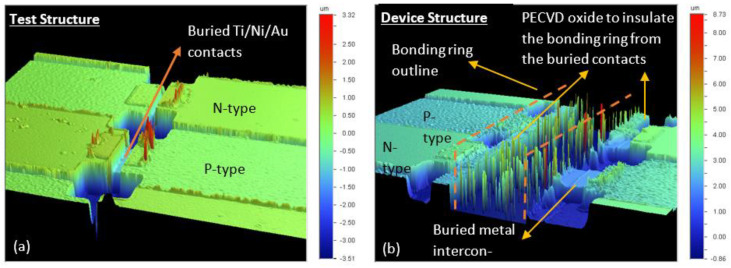
(**a**) 3D profile of a test structure without a bonding ring between each thermocouple-like structure. (**b**) The device structure’s 3D profile illustrates the PECVD oxide on which the bonding ring is deposited to insulate it from the buried metal interconnects connecting the thin films on either side of the bonding ring.

As shown in Figure 15, the cross-section schematic displayed in Figure 11 is achievable via the fabrication process outlined in this work. We note that the vacuum cavities have not yet been integrated into the device wafers. To highlight the buried metal interconnects and contrast the challenges of creating bonding rings, a test structure from a non-device sample is presented in Figure 15a.

As we proceed to the packaging steps involving wafer bonding and deep reactive ion etching, the thermoelectric thin films will be exposed to temperatures of 200–300 °C, which may create additional changes to their power factors. Therefore, tests are being conducted on low temperature (200 °C) Au-In eutectic bonding to provide a low thermal budget option to the Au-Sn bonding, which requires 300 °C.

## 4. Conclusions

Results were presented on developing bismuth antimony telluride and bismuth selenium telluride thin films to fabricate a hybrid µ-TEG via MEMS-compatible processes. Thermoelectric properties of the thin films were optimized by tuning the pellet compositions and the post-deposition baking temperatures. It was found that power factors of 2.75 mW m^−1^ K^−2^ and 0.59 mW m^−1^ K^−2^ could be attained by evaporating at room temperature from pellets with compositions Bi_0.5_Sb_1.5_Te_3_ (Te4x) and Bi_2_Se_0.3_Te_2.7_ (Se9xTe2x), respectively. Films were deposited via lift-off. The selection of adhesion layers between the film and the substrate was found to be crucial for the device fabrication and film properties. It was observed that a ~15 nm Cr adhesion layer with p-type thin films on a Si/SiO_2_ substrate led to unexpected n-type behavior during the thermoelectric measurements. Consequently, MPTMS and Cr were identified as optimum adhesion layers for p-type and n-type thin films. The pre-packaging device fabrication steps involving wafer bonding ring formation and micro-patterning of thermoelectric thin films were optimized on a 4 wafer. Results from device wafer fabrication revealed the trade-offs between adhesion layers, lift-off, substrate selection, and thermoelectric performance.

## Figures and Tables

**Table 1:** Melting points and vapor pressures [42] of the constituent elements of the pellets and thermoelectric thin films.

Material	Melting Point	Vapor Pressure
Bi	271.7 °C	0.76 Torr at 893 °C
Te	449.7 °C	0.76 Torr at 505 °C
Sb	630.8 °C	1 Torr at 886 °C
Se	217 °C	0.76 Torr at 344 °C

**Table 2:** Data to demonstrate the variation in the compositions of different n-type pellets.

Sample Name	Weight of Each Element (g)			Total Weight(g)
	Bi	Se	Te	
Bi_2_Se_0.3_Te_2.7_ (standard)	5.3164	0.3013	4.3823	10.0000
Bi_2_Se_0.3_Te_2.7_ (Se4x, Te2x)	5.3164	1.2052	8.7646	15.2862
Bi_2_Se_0.3_Te_2.7_ (Se9x, Te1x)	5.3164	2.7117	4.3823	12.4104

**Table 3:** Thermoelectric properties from the set of samples with optimum pellet compositions and bake times but deposited with different adhesion layers on three substrates.

Run ID	Pellet Composition	Anneal	Substrate	Adhesion Layer	At Room Temperature			At 150 °C		
					S (µV/K)	σ (S/cm)	Power Factor (mWK^−2^m^−1^)	S (µV/K)	σ (S/cm)	Power Factor (mWK^−2^m^−1^)
1r	Te4x, (p-type)	270 °C for 24 h	Glass	Cr (15 nm)	189.4	281.0	1.01	219.5	267.1	1.28
			Si/SiO_2_		−57.9	476.6	0.16	−136.5	579.7	1.08
			High ρ Si		150.8	190.0	0.43	168.1	193.6	0.55
1s	Te4x, (p-type)	270 °C for 24 h	Glass	MPTMS	228.5	355.0	1.81	215.3	273.6	1.20
			Si/SiO_2_		177.9	314.8	0.99	189.5	246.8	0.89
			High ρ Si		160.3	273.6	0.70	175.8	218.1	0.67
1t	Te4x, (p-type)	270 °C for 24 h	Glass	None	231.9	510.5	2.75	223.9	373.4	1.87
			Si/SiO_2_		234.9	475.9	2.63	203.2	363.2	1.50
			High ρ Si		210.5	479.7	2.12	212.1	332.7	1.52
2p	Se9x, Te2x (n-type)	360 °C for 24 h	Glass	Cr (15 nm)	−158.7	233.6	0.59	−170.2	195.8	0.57
			Si/SiO_2_		−106.2	308.2	0.35	−158.8	285.6	0.72
			High ρ Si		−127.6	223.3	0.36	−126.9	191.1	0.31
2q			Glass	MPTMS	−129.6	263.2	0.44	−139.3	215.3	0.42
			Si/SiO_2_		−80.7	247.9	0.16	−49.0	242.5	0.06
			High ρ Si		−117.4	273.6	0.38	−136.3	223.1	0.42

**Table 4:** Comparison of this work with other studies on near room temperature deposition of Bismuth Telluride thin films using evaporation and sputtering based methods.

Year	Method	Substrate **	Post-Deposition Anneal	p-Type			n-Type			Micropatterning
				S (µV/K)	PF ^■^	PF ^▲^, T(K)	S (µV/K)	PF ^■^	PF ^▲^, T(K)	
2005 [21]	CE *	Si heated to 403 K	-	97	0.30	NA	−74	0.15	NA	Omnicoat/SU-8 mold for lift-off
2008 [34]	DC-MS	Glass	523 K for 16–32 h in Ar	191	1.6	NA	NA	1.2	NA	-
2013 [46]	RF-MS *	SiO_2_ glass	573 K for 1 h under vacuum	~190	3.61	4.0 (450K)	~−180	3.25	2.25 (450K)	PMER PCA-1000PM photoresist for lift-off
2018 [52]	RF-MS *	Glass	None	NA	NA	NA	−90	0.3	NA	-
2020 [53]	RF-MS *	Glass	573 K for 1 h in Ar(95%) +H(5%)	NA	NA	NA	−150	1.65	NA	-
2021 [54]	RF-MCS *	2” Si/SiO_2_ wafer	523 K for 30 min in N_2_	110	1.3	NA	−102	0.7	NA	15um AZ9620 resist for lift-off
2021 [55]	RF-MS *	Pre-structured measurement chip	523 K for 4 h in Ar	NA	NA	NA	−103	0.51	0.59 (473K)	Shadow mask
2020 [56]	2-step SS-TE *	Polyimide	RHT ^#^ from 300 K to 500 K at 4 K/s in vacuum	NA	NA	NA	−126	0.45	0.53 (450K)	-
This work	SS-TE *	Glass	543 K for 24 h for p-type 633 K for 24 h for n-type (in Argon)	231	2.75	1.87 (423K)	−158	0.59	0.57 (423K)	Image reversed AZ4620 photoresist for lift-off
		4” Si/SiO_2_ wafer		177	0.99	0.99 (300K)	−106	0.35	1.54 (491K)	

* CE: co-evaporation; RF-MS: RF magnetron sputtering; RF-MCS: RF magnetron co-sputtering; SS-TE: single source thermal evaporation; FE: flash evaporation; DC-MS: DC magnetron sputtering. ** Room temperature deposition unless stated otherwise. **^■^** PF: Power factor (mWm^−1^K^−2^) at room temperature. ^▲^ PF: Maximum power factor (mWm^−1^K^−2^) and the corresponding temperature. NA: The data are reported only at room temperature. ^#^ Rapid heat treatment.

## Data Availability

Data is available upon request.
